# Facile Preparation of a Plasmon-Enhanced Ag-CuO/TiO_2_ for the Efficient Visible-Light-Driven Photodegradation of Tetracycline Hydrochloride

**DOI:** 10.3390/ma19112189

**Published:** 2026-05-22

**Authors:** Lianmin Cui, Li Ren, Zhi Chen, Benfeng Zhu, Chen Xu, Guoying Wei

**Affiliations:** College of Materials and Chemistry, China Jiliang University, Hangzhou 310018, China; p23050854010@cjlu.edu.cn (L.C.);

**Keywords:** heterostructure, tetracycline hydrochloride, silver nanoparticles, visible light, photodegradation

## Abstract

Water pollution caused by antibiotics is a growing problem. Therefore, photodegradation by efficient catalysts is an environmentally friendly technology that can effectively degrade organic pollutants in water. In this work, a method was innovatively used to prepare a ternary heterostructure of plasmon-enhanced Ag-CuO/TiO_2_. The composite was synthesized through a facile stepwise strategy involving the formation of CuO nanorods, TiO_2_ coating, and subsequent deposition of Ag nanoparticles on their surface using AgNO_3_, enabling intimate interfacial contact among the different components. The prepared samples were characterized by XRD, HRTEM, XPS, and UV-Vis. The chemical composition of the composite Ag-CuO/TiO_2_ showed a Cu/Ti atomic ratio of 2.58, as well as a Ag/Cu ratio of 0.91. The UV-Vis spectrum reveals the largest absorption peak at 550 nm for the composite Ag-CuO/TiO_2_. The prepared Ag-CuO/TiO_2_ composites were applied to the visible-light degradation of tetracycline hydrochloride, with the photocatalytic degradation rate reaching 80.7% under the optimal conditions within 60 min, which is significantly better than CuO and CuO/TiO_2_ without silver nanoparticles. Capture experiments indicated that h^+^ are involved during the course of the photodegradation and that h^+^ are the main active substances. Furthermore, the proposed mechanism for the photodegradation of the Ag-CuO/TiO_2_ composites is given. It has potential applications in the treatment of organic pollutants in water.

## 1. Introduction

The presence of persistent organic pollutants in water has become an increasing environmental concern, particularly for compounds that are resistant to natural degradation. Among these, antibiotics are frequently detected due to their widespread consumption and incomplete removal during conventional wastewater treatment. Tetracycline hydrochloride (TCH) is a representative example, known for its stable molecular structure and poor biodegradability, which make it difficult to eliminate using traditional treatment technologies. In this work, semiconductor photocatalysis driven by visible light has gained attention as an alternative approach capable of transforming such recalcitrant contaminants [1].

Titanium dioxide (TiO_2_) is one of the most extensively studied photocatalysts due to its chemical stability, low toxicity and low cost [2,3]. Nevertheless, the large band gap of TiO_2_ restricts its optical response primarily to the ultraviolet region, and the rapid recombination of photogenerated charge carriers further restricts its photocatalytic efficiency. Forming composite structures with narrow-band gap semiconductors has therefore become a common strategy to improve visible-light utilization and promote charge separation [4,5].

Copper(II) oxide (CuO), a p-type semiconductor with a relatively narrow band gap, has been widely explored for visible-light photocatalysis [6,7]. When coupled with TiO_2_, the formation of a p-n heterojunction can facilitate the separation of photogenerated carriers and drive their migration across the interface. However, many reported CuO/TiO_2_ systems still suffer from limited interfacial charge-transfer efficiency, and carrier recombination remains a key factor restricting overall activity. These observations indicate that additional regulation of interfacial charge behavior is necessary to further improve the performance of such binary heterostructures [8,9,10].

Surface modification with metallic nanoparticles offers a further means of influencing interfacial electronic processes [11,12]. Silver (Ag) is of particular interest because of its ability to interact electronically with adjacent semiconductors and its role in visible-light response. Metallic Ag can function as an electron mediator, capturing photogenerated electrons and assisting their migration, which may help to suppress recombination and accelerate surface redox reactions. Introducing Ag into a CuO/TiO_2_ system is therefore expected to provide additional pathways for charge separation beyond those available in the binary junction alone [13,14].

Based on these considerations, a ternary Ag-CuO/TiO_2_ photocatalyst was constructed in the present work with the aim of improving the visible-light-driven degradation of TCH. CuO nanorods were used as the primary visible-light-absorbing component, TiO_2_ was incorporated to form a heterojunction structure, and Ag nanoparticles were subsequently deposited to modify interfacial charge transfer. The materials were characterized in terms of their structure, optical properties and photoelectrochemical behavior, and their photocatalytic activities were evaluated through the degradation of TCH under visible-light irradiation. Emphasis was placed on understanding how the interfacial configuration influences photocatalytic performance [15,16,17,18].

## 2. Materials and Methods

### 2.1. Materials

Chemicals including copper(II) chloride dihydrate (CuCl_2_·2H_2_O, AR), tetrabutyl titanate (TBT, AR), sodium hydroxide (NaOH, 96%), sodium citrate (C_6_H_5_Na_3_O_7_·2H_2_O, AR), polyvinylpyrrolidone ((C_6_H_9_NO)_n_, PVP, average Mw ≈ 8000, K16-K18, AR), silver nitrate (AgNO_3_, AR), Nafion (D520, 5 wt.% in a mixture of lower aliphatic alcohols), tert-butanol (C_4_H_10_O, TBA, >99.0%, AR), ethylenediaminetetraacetic acid disodium salt dihydrate (C_10_H_14_N_2_Na_2_O_8_·2H_2_O, EDTA-2Na, 98%, AR), sodium sulfate (Na_2_SO_4_, AR), tetracycline hydrochloride (C_22_H_24_N_2_O_8_·HCl, TC-HCl, BR), and L-ascorbic acid (C_6_H_8_O_6_, >99.0%, AR) were purchased from Macklin Chemical Co., Ltd. (Shanghai, China). Ammonia solution (NH_3_·H_2_O, 25–28%), 2,2,6,6-tetramethylpiperidinooxy (C_9_H_18_NO, TEMPO, 98%, AR), and anhydrous ethanol (C_2_H_6_O, 95%) were purchased from Hangzhou Gaojing Fine Chemical Co., Ltd. (Hangzhou, China). Deionized water was used throughout all experiments. All chemicals were used as received without further purification.

### 2.2. Preparation Method

#### 2.2.1. Synthesis of CuO Nanorods

CuO nanorods were prepared via an alkaline precipitation method [19,20,21]. First, 2.40 g of sodium hydroxide and 3.53 g of sodium citrate were dissolved in 150 mL of deionized water under continuous stirring in a 250 mL round-bottom flask. Subsequently, 0.40 g of polyvinylpyrrolidone (PVP) and 2.50 g of CuCl_2_·2H_2_O were added to the solution, and the mixture was stirred until a homogeneous solution formed. The reaction mixture was then maintained at 90 °C in an oil bath for 2 h. After naturally cooling to room temperature, the black precipitate was collected by centrifugation, washed three times with deionized water and ethanol, and dried at 60 °C for 12 h. Finally, the obtained precursor was calcined at 350 °C for 2 h to improve crystallinity and ensure complete conversion to CuO.

#### 2.2.2. Preparation of CuO/TiO_2_ Composite

The CuO/TiO_2_ composite was synthesized via an ethanol–ammonia-assisted sol–gel method [22,23]. First, 0.35 g of CuO nanorods and 0.35 g of polyvinylpyrrolidone (PVP) were dispersed in 100 mL of ethanol under continuous stirring. Subsequently, 0.33 mL of ammonia solution (NH_3_·H_2_O, 25 wt.%) and 0.20 mL of deionized water were added to regulate the hydrolysis environment. The suspension was ultrasonicated for 30 min and then stirred for an additional 30 min. Afterward, 1.00 mL of tetrabutyl titanate (TBT) was added dropwise, and the mixture was maintained at 45 °C for 12 h to allow controlled hydrolysis and gel formation. The resulting solid was collected by centrifugation, washed with deionized water and ethanol, and dried at 60 °C for 12 h. Finally, the sample was calcined stepwise at 200 °C for 60 min, 350 °C for 60 min, and 450 °C for 120 min with a heating rate of 5 °C min^−1^, yielding the CuO/TiO_2_ heterojunction. The selected calcination temperatures were performed based on our previous experiments. The stepwise thermal treatment was found to effectively remove the residual solvents and organic species while ensuring the formation of the desired crystalline structure.

#### 2.2.3. Preparation of Ag-CuO/TiO_2_ Ternary Composite

**Figure 1:**
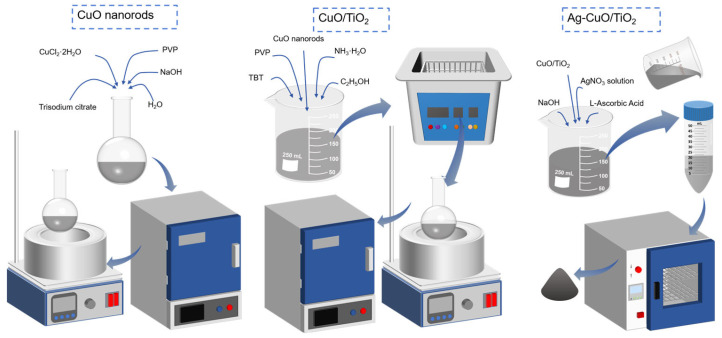
Preparation schematic of Ag-CuO/TiO_2_ composites.

Ag nanoparticles were deposited onto the CuO/TiO_2_ composite via an in situ chemical reduction method [24,25,26]. First, 0.05 g of CuO/TiO_2_ powder was dispersed in 10 mL of deionized water under continuous stirring. Subsequently, 800.00 μL of AgNO_3_ solution (0.10 g mL^−1^) was added, and the mixture was stirred for 60 min to allow adsorption of Ag^+^ ions onto the surface of the composite. The pH of the suspension was then adjusted to approximately 7.0 using 0.10 M NaOH solution. The suspension was centrifuged at 4500 rpm for 20 min, and the collected solid was redispersed in 20 mL of deionized water. Afterward, 0.09 g of L-ascorbic acid was added as a reducing agent, and the mixture was stirred for 30 min to complete the in situ reduction in Ag^+^. The final product was washed several times with deionized water and ethanol and dried at 100 °C for 12 h, yielding the Ag-CuO/TiO_2_ composite. A schematic diagram of the synthesis process of Ag-CuO/TiO_2_ is shown in Figure 1.

### 2.3. Sample Characterization

The crystal structure of the Ag-CuO/TiO_2_ composite was characterized by X-ray diffraction (XRD) using a Rigaku Ultima IV diffractometer (Tokyo, Japan) with Cu Kα radiation (λ = 0.154178 nm) monochromated by graphite. The diffraction patterns were recorded in the 2θ range of 10–90° at a scanning rate of 5° min^−1^. The morphology and microstructure of the samples were examined by scanning electron microscopy (SEM, Hitachi S-4800, Tokyo, Japan) equipped with energy-dispersive X-ray spectroscopy (EDS). The elemental composition of the samples was quantitatively determined by inductively coupled plasma optical emission spectroscopy (ICP-OES) after acid digestion. Transmission electron microscopy (TEM) and high-resolution TEM (HRTEM) observations were performed on a JEM-2100F microscope (JEOL, Tokyo, Japan) operated at an accelerating voltage of 200 kV. The surface elemental composition and chemical states were analyzed by X-ray photoelectron spectroscopy (XPS) using a Thermo Scientific K-Alpha spectrometer (Waltham, MA, USA) with monochromatic Al Kα radiation. The specific surface area and pore structure of the samples were analyzed by N_2_ adsorption–desorption measurements. The Brunauer–Emmett–Teller (BET) method was used to calculate the specific surface area, and the pore size distribution was analyzed over a wide pore size range. The optical absorption properties were measured using a Shimadzu UV-3600 spectrophotometer (Kyoto, Japan) equipped with an integrating sphere. The optical band gaps of the samples were estimated from Tauc plots based on the relation (αhυ)^2^ = A(hυ − E_g_), where α is the absorption coefficient, h is Planck’s constant, υ is the frequency of light, A is a constant, and E_g_ is the optical band gap. Here, n = 2, corresponding to a direct allowed transition.

### 2.4. Photoelectrochemical Measurements

Photocatalytic degradation experiments were conducted under visible-light irradiation using a 300 W Xe lamp equipped with a cutoff filter (λ > 400 nm) as the light source. In a typical run, 10 mg of catalyst was dispersed in 50 mL of tetracycline hydrochloride (TC-HCl, 10 mg/L) solution in a beaker. Prior to irradiation, the suspension was magnetically stirred in the dark for 60 min to establish adsorption–desorption equilibrium. During the reaction, 3.5 mL aliquots were withdrawn at 10 min intervals and centrifuged to remove the catalyst. The concentration of TC-HCl was determined from the absorbance at 357 nm using a UV-2600 spectrophotometer (Shimadzu, Kyoto, Japan). The degradation efficiency (DR, %) of TC-HCl was calculated according to the following equation:

$$\mathrm{DR}\;=\;\left(1\;-\;\frac{A_{i}}{A_{0}}\right)\times \;100\%$$

In the above equation, A_i_ represents the absorbance of the sample at a given irradiation time, while A_0_ corresponds to the initial absorbance of the solution after adsorption–desorption equilibrium was established.

To further investigate the kinetics of the photocatalytic reaction, the experimental degradation data were fitted using a pseudo-first-order kinetic model:

$$\ln(\frac{C_{0}}{C})\;=\;\mathrm{kt}$$

In this equation, k is the apparent first-order rate constant (min^−1^), C_0_ represents the initial concentration of TC-HCl after adsorption–desorption equilibrium was reached, and C denotes the instantaneous concentration of TC-HCl at irradiation time t.

Reusability of the photocatalyst was evaluated through consecutive degradation cycles. An initial amount of 10 mg of catalyst was added to the reaction system, and the removal efficiency of the target pollutant was determined after 60 min of light irradiation. After each run, a concentrated pollutant solution (100 mg/L) was added to restore the pollutant concentration to the level after the dark adsorption–desorption equilibrium, and the subsequent cycle was carried out under identical conditions without adding additional catalyst. During the reaction, 3.5 mL aliquots were withdrawn at 10 min intervals for absorbance analysis. The degradation efficiencies over successive cycles were compared to assess the stability and reusability of the catalyst.

To gain insight into the photocatalytic mechanism, radical scavenging experiments were carried out. Specifically, tert-butanol (TBA, 2 mM), 2,2,6,6-tetramethylpiperidine-N-oxyl (TEMPO, 2 mM), and ethylenediaminetetraacetic acid disodium salt (EDTA-2Na, 2 mM) were employed as scavengers for hydroxyl radicals (•OH), superoxide radicals (•O_2^−^_), and photogenerated holes (h^+^), respectively. The degradation tests were then performed under the same conditions, and the dominant reactive species were identified based on the corresponding changes in absorbance.

Photoelectrochemical measurements were conducted on a CHI660E electrochemical workstation using a conventional three-electrode system, with a platinum wire as the counter electrode and an Ag/AgCl electrode as the reference electrode. The working electrode was prepared by dispersing 10 mg of catalyst in 1.5 mL of Nafion solution, followed by spin-coating the suspension onto FTO conductive glass (1 cm × 1 cm). A 0.5 M Na_2_SO_4_ aqueous solution served as the electrolyte. Visible-light irradiation was provided by a 300 W Xe lamp equipped with a cutoff filter (λ > 400 nm). Transient photocurrent responses were recorded under intermittent light irradiation with 20 s light on/off cycles, and electrochemical impedance spectroscopy (EIS) measurements were performed over a frequency range of 0.01 Hz to 100 kHz with an AC perturbation amplitude of 5 mV.

## 3. Results

### 3.1. Microstructural Analysis of Ag-CuO/TiO_2_ Composites

**Figure 2:**
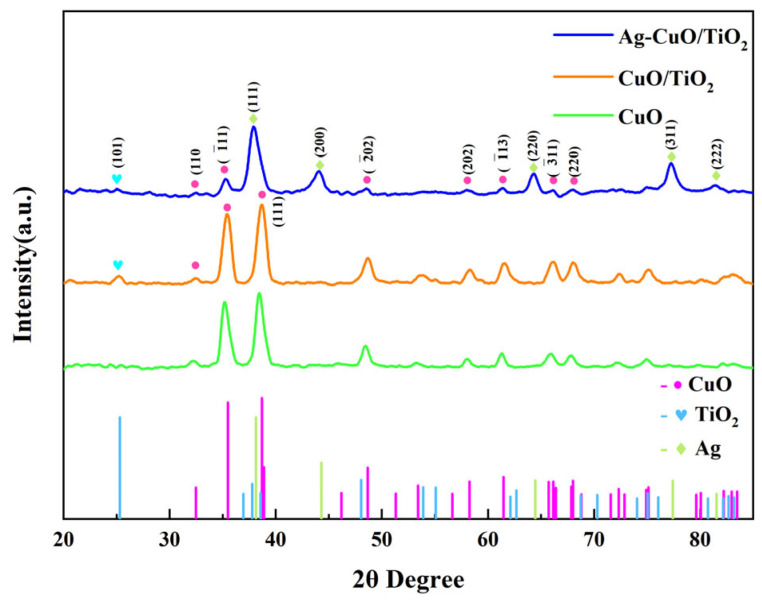
XRD patterns of pure CuO, CuO/TiO_2_ and Ag-CuO/TiO_2_ composites.

The Ag-CuO/TiO_2_ composites were prepared via a facile stepwise strategy, including the formation of CuO nanorods, subsequent TiO_2_ coating, and in situ reduction of Ag nanoparticles on the surface using AgNO_3_. Powder X-ray diffraction (XRD) was performed to investigate the crystal structures of CuO, CuO/TiO_2_, and Ag-CuO/TiO_2_ nanostructures. The corresponding XRD patterns are shown in Figure 2.

The X-ray diffraction (XRD) patterns of CuO, CuO/TiO_2_, and Ag-CuO/TiO_2_ are shown in Figure 2. For the pure CuO sample, the diffraction peaks at 2θ ≈ 35.5°, 38.7°, 48.7°, 53.4°, 58.3°, 61.5°, 66.1°, and 68.0° can be indexed to the ($\bar{1}$11), (111), ($\bar{2}$02), (020), (202), ($\bar{1}$13), ($\bar{3}$11), and (220) planes of monoclinic CuO (JCPDS No. 04-007-0518) [20,21,27,28,29,30], indicating high crystallinity and phase purity. In the CuO/TiO_2_ composite, additional peaks appear at 2θ ≈ 25.3°, 37.8°, 48.0°, 53.9°, and 55.1°, which correspond to the (101), (004), (200), (105), and (211) planes of anatase TiO_2_ (JCPDS No. 04-002-2750) [31,32], while the characteristic reflections of CuO remain unaltered, suggesting that the CuO crystal structure is preserved during composite formation. For the Ag-CuO/TiO_2_ sample, new diffraction peaks are observed at 2θ ≈ 38.1°, 44.3°, 64.4°, 77.4°, and 81.5°, which can be assigned to the (111), (200), (220), (311), and (222) planes of face-centered cubic metallic Ag (JCPDS No. 04-001-2617) [33,34]. No peaks corresponding to impurities were detected, indicating the successful coexistence of CuO, TiO_2_, and Ag in the composite while maintaining their respective crystalline phases.

**Figure 3:**
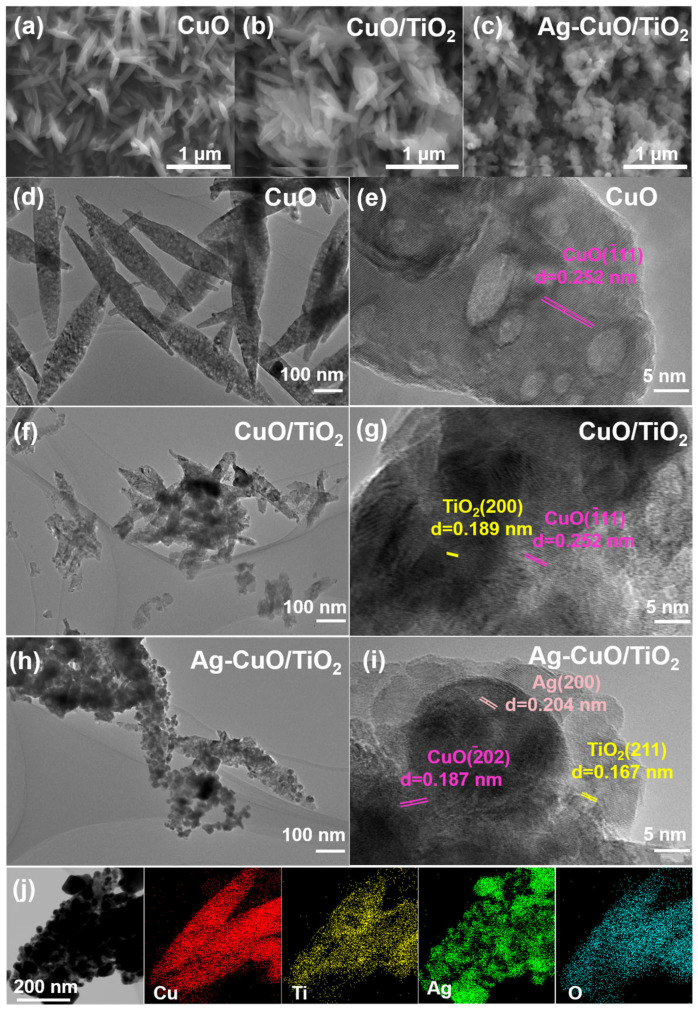
(**a**–**c**) SEM images of CuO, CuO/TiO_2_ and Ag-CuO/TiO_2_; (**d**–**i**) TEM images of CuO, CuO/TiO_2_ and Ag-CuO/TiO_2_; (**j**) elemental mapping of Ag-CuO/TiO_2_ (Cu, Ti, O and Ag).

The morphologies and microstructures of CuO, CuO/TiO_2_ and Ag-CuO/TiO_2_ were investigated by SEM and TEM, with the results shown in Figure 3. As displayed in Figure 3a, the pristine CuO sample consists predominantly of well-dispersed spindle-like nanorods with tapered ends and relatively smooth surfaces. After coupling with TiO_2_, the overall rod-like morphology is largely maintained; however, numerous fine nanoparticles can be observed decorating the surface of the CuO nanorods (Figure 3b), indicating the formation of a TiO_2_ coating layer and the establishment of interfacial contact between the two semiconductors. Following Ag deposition, additional smaller particles appear on the surface of the composite (Figure 3c), leading to a rougher texture and suggesting the successful loading of Ag nanoparticles onto the CuO/TiO_2_ framework [35].

TEM observations further confirm the structural features of the samples. As shown in Figure 3d,e, pristine CuO exhibits a well-defined nanorod morphology with clear lattice fringes, and an interplanar spacing of approximately 0.252 nm can be assigned to the (111) plane of CuO. After coupling with TiO_2_, the rod-like morphology is largely retained (Figure 3f); however, the surface becomes rougher due to the attachment of TiO_2_ nanoparticles, accompanied by a certain degree of particle aggregation. The high-resolution TEM image (Figure 3g) shows distinct lattice fringes with spacings of about 0.252 nm and 0.189 nm, corresponding to the (111) plane of CuO and the (200) plane of TiO_2_, respectively, indicating the formation of a CuO/TiO_2_ heterojunction. Upon further introduction of Ag, additional nanoparticles are observed on the surface of the composite (Figure 3h). In the HRTEM image (Figure 3i), lattice fringes with spacings of approximately 0.187 nm, 0.204 nm, and 0.167 nm can be identified and are reasonably assigned to the ($\bar{2}$02) plane of CuO, the (200) plane of metallic Ag, and the (211) plane of TiO_2_, respectively. Slight lattice distortions are observed at the interfaces between different phases, suggesting lattice mismatch and the formation of closely contacted heterointerfaces, which are favorable for interfacial charge transfer [14,15,36].

Elemental mapping analysis of Ag-CuO/TiO_2_ (Figure 3j) demonstrates that Cu, Ti, O, and Ag are homogeneously distributed within the composite. The EDS results (Table S1) provide a semi-quantitative indication of the elemental composition [37]. The measured elemental contents (Cu: 28.03 at.%, Ag: 24.43 at.%, Ti: 7.63 at.%, O: 39.92 at.%) confirm the successful incorporation of these elements. The deviation from the nominal ratios may be attributed to the surface-sensitive nature and semi-quantitative limitation of EDS analysis. To obtain a more accurate determination of the bulk elemental composition, inductively coupled plasma optical emission spectrometry (ICP-OES) was further performed. The measured elemental composition (Cu: 27.78 wt.%, Ag: 43.03 wt.%, Ti: 8.07 wt.%) is summarized in Table 1. The calculated Cu/Ti atomic ratio is approximately 2.58, while the Ag/Cu ratio is about 0.91, and the results are in good agreement with the designed compositions. Furthermore, N_2_ adsorption–desorption measurements were carried out to investigate the specific surface area and pore structure of the samples. All samples exhibit typical type IV isotherms, indicating the presence of mesoporous structures. The corresponding specific surface areas and pore parameters are provided in Figure S1 (Supplementary Materials).

**Figure 4:**
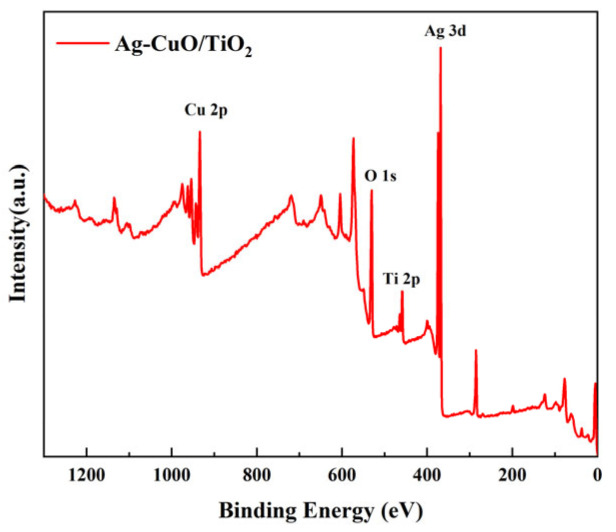
XPS survey spectrum of Ag-CuO/TiO_2_.

**Figure 5:**
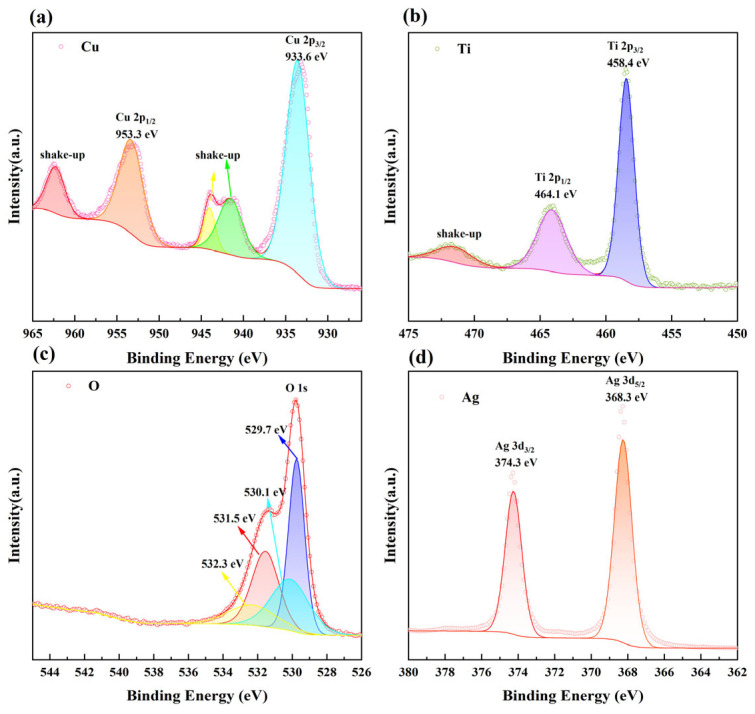
High-resolution XPS spectra of (**a**) Cu 2p, (**b**) Ti 2p, (**c**) O 1s and (**d**) Ag 3d for the Ag-CuO/TiO_2_ composite.

The surface elemental composition and chemical states were further analyzed by XPS, as shown in Figure 4 and Figure 5, and all binding energies were calibrated against the C 1s peak at 284.8 eV. The survey spectrum confirms the presence of Cu, Ti, O and Ag, consistent with the XRD results. In the high-resolution Cu 2p spectrum shown in Figure 5a, two main peaks located at 933.6 and 953.5 eV are attributed to Cu 2p_3/2_ and Cu 2p_1/2_ of Cu^2+^, accompanied by characteristic shake-up satellite peaks, indicating that copper predominantly exists in the CuO state [38,39,40]. As shown in Figure 5b, the Ti 2p spectrum displays peaks at 458.4 and 464.1 eV, corresponding to Ti 2p_3/2_ and Ti 2p_1/2_ of Ti^4+^ with a spin–orbit splitting of 5.7 eV, which is consistent with TiO_2_ and shows no evidence of Ti^3+^ species [41,42,43,44]. As shown in Figure 5c, The O 1s spectrum can be deconvoluted into components assigned to lattice oxygen (~529.7 eV), oxygen associated with defect sites (~530.1 eV), and surface hydroxyl or adsorbed oxygen species (531.5–532.3 eV), suggesting the presence of reactive surface oxygen species [45,46,47]. In the Ag 3d spectrum shown in Figure 5d, peaks centered at 368.3 and 374.3 eV correspond to Ag 3d_5/2_ and Ag 3d_3/2_ of metallic Ag^0^, with no detectable signals from oxidized silver species, confirming that Ag is present in its metallic state on the composite surface. The coexistence of CuO, TiO_2_ and metallic Ag, together with their stable chemical states, provides a structural basis for interfacial charge transfer within the ternary composite system [48,49,50]. In addition, XPS analysis indicates that the Ag-CuO/TiO_2_ sample exhibits a Cu/Ti/Ag/O atomic ratio of approximately 10:1:12:7, confirming the successful incorporation of Ag onto the CuO/TiO_2_ composite.

**Figure 6:**
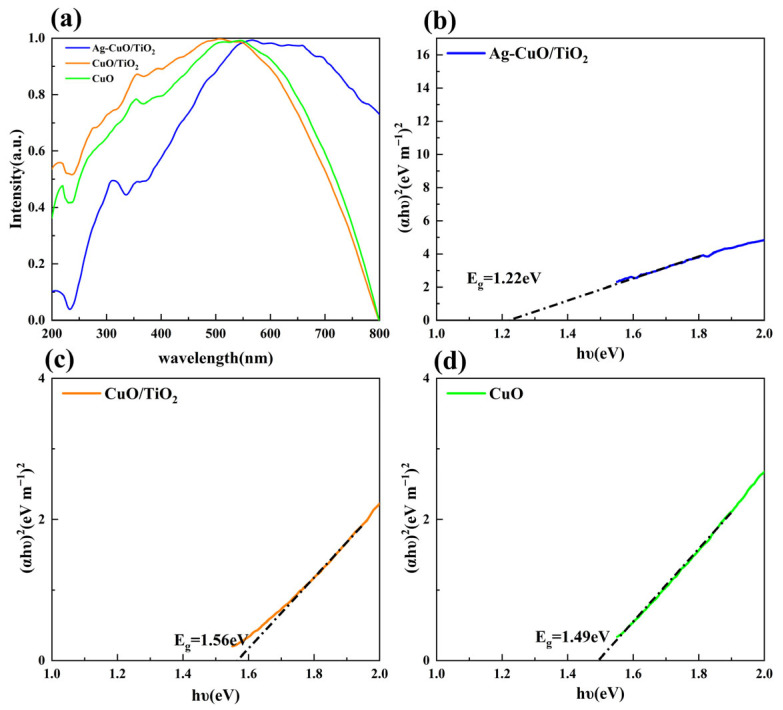
Optical absorption and photoelectrochemical properties of CuO, CuO/TiO_2_ and Ag-CuO/TiO_2_: (**a**) UV-Vis DRS spectra; (**b**–**d**) Tauc plots.

The UV-Vis diffuse reflectance spectra of CuO, CuO/TiO_2_, and Ag-CuO/TiO_2_ catalysts, measured over the 200–800 nm range, are shown in Figure 6a. The maximum absorption peaks occur at approximately 500 nm for CuO, 450–480 nm for CuO/TiO_2_, and 550 nm for Ag-CuO/TiO_2_. Pure CuO exhibits strong absorption across the visible region, with a relatively weak response in the ultraviolet range. Coating with TiO_2_ slightly enhances absorption below 400 nm, reflecting the intrinsic optical properties of TiO_2_, while the visible-light absorption profile remains similar to that of CuO. In contrast, Ag-CuO/TiO_2_ displays a markedly extended absorption range from 400 to 800 nm, with a pronounced peak at 550 nm, which can be attributed to the localized surface plasmon resonance (LSPR) of Ag nanoparticles. These results indicate that the incorporation of Ag significantly improves the visible-light harvesting capability of the composite, rendering Ag-CuO/TiO_2_ a promising candidate for visible-light-driven photocatalysis [36,37].

In addition, the optical band gaps of the samples were estimated from Tauc plots (Figure 6b–d) using the relation (αhυ)^2^ = A(hυ − E_g_) [11,29,51,52,53]. The calculated band gaps are approximately 1.49 eV for CuO, 1.56 eV for CuO/TiO_2_, and 1.22 eV for Ag-CuO/TiO_2_. The slightly wider band gap of CuO/TiO_2_ reflects the contribution of the wide-band gap TiO_2_, whereas the narrower band gap of Ag-CuO/TiO_2_ indicates that the incorporation of Ag modifies the interfacial electronic structure, reducing the forbidden bandwidth and facilitating photoexcitation. The narrower band gap thus enhances the photocatalytic activity of Ag-CuO/TiO_2_ [31,54].

### 3.2. Catalytic Performane

**Figure 7:**
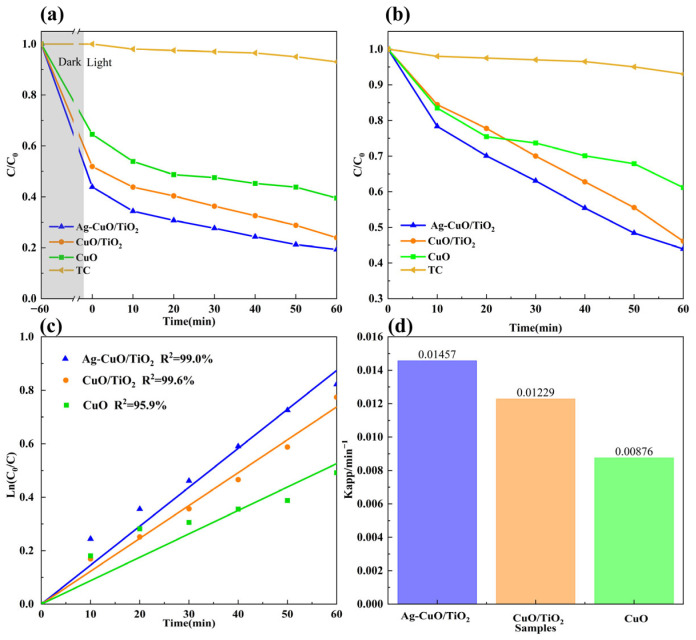
Photocatalytic degradation of TCH over CuO, CuO/TiO_2_ and Ag-CuO/TiO_2_: (**a**,**b**) degradation curves; (**c**) kinetic fitting plots; (**d**) rate constants.

The degradation ability of the prepared photocatalysts was examined using tetracycline hydrochloride as the target pollutant. The visible-light photocatalytic degradation of the tetracycline hydrochloride (TCH) over CuO, CuO/TiO_2_ and Ag-CuO/TiO_2_ is shown in Figure 7. During the initial dark stage, all samples exhibited a certain degree of TCH adsorption, indicating the establishment of adsorption–desorption equilibrium on the catalyst surfaces. After illumination, the TCH concentration decreased continuously with reaction time [53,55].

**Figure 8:**
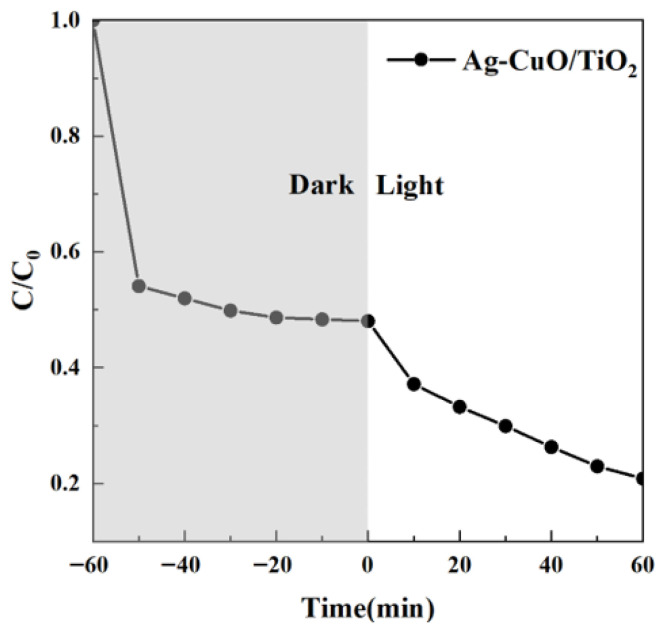
Dark adsorption stage curve of Ag-CuO/TiO_2_.

As shown in Figure 7a, under catalyst-free conditions, the concentration of tetracycline hydrochloride remains nearly unchanged after 60 min of visible-light irradiation, indicating negligible photolysis in the system. In contrast, pure CuO exhibits the lowest removal performance, with a total removal efficiency of only 60.5% after 60 min. The introduction of TiO_2_ improves the removal efficiency to 76.1% for the CuO/TiO_2_ composite. Upon further incorporation of Ag nanoparticles, the removal efficiency of Ag-CuO/TiO_2_ increases to 80.7%, demonstrating that the synergistic effects of TiO_2_ and Ag contribute to enhanced photocatalytic performance. After the dark adsorption stage, the adsorption efficiencies of Ag-CuO/TiO_2_, CuO/TiO_2_, and pure CuO are calculated to be 56.1%, 48.1%, and 35.4%, respectively, indicating that the introduction of TiO_2_ and Ag enhances the adsorption capacity of the catalyst. To further distinguish the contributions of adsorption and photocatalytic degradation, the changes during the irradiation stage are analyzed separately, as shown in Figure 7b. After 60 min of visible-light irradiation, the actual photocatalytic degradation efficiencies of Ag-CuO/TiO_2_, CuO/TiO_2_, and pure CuO are determined to be 56.1%, 53.9%, and 38.8%, respectively. Although the adsorption efficiency of Ag-CuO/TiO_2_ is comparable to its photocatalytic degradation efficiency, the enhanced adsorption facilitates the enrichment of tetracycline molecules on the catalyst surface, thereby promoting subsequent photocatalytic reactions rather than dominating the overall removal process. The UV-Vis absorption spectra of tetracycline over Ag-CuO/TiO_2_ at different irradiation times under visible light are shown in Figure S2 (Supplementary Materials), confirming efficient removal of pollutants. The degradation of tetracycline hydrochloride by all the photocatalysts follows a first-order kinetic model. Figure 7c presents the corresponding kinetic fitting curves, while the calculated rate constants are summarized in Figure 7d. The rate constants are determined to be 0.01457 min^−1^ for Ag-CuO/TiO_2_, 0.01229 min^−1^ for CuO/TiO_2_, and 0.00876 min^−1^ for CuO [49,56], with high correlation coefficients (R^2^) of 99.0%, 99.6%, and 95.9%, respectively, indicating good fitting quality. In addition, the adsorption portion of the concentration curves is provided in Figure 8, which shows that after 30 min of the dark stage the solution of the tetracycline hydrochloride reaches adsorption–desorption equilibrium. The Ag-CuO/TiO_2_ photocatalyst developed in this work exhibits superior photocatalytic performance compared with previously reported Nb_2_O_5_, ZnO, TiO_2_, and Au-Nb_3_O_7_F systems [57]. A summary of the degradation efficiencies is presented in Table 2.

**Figure 9:**
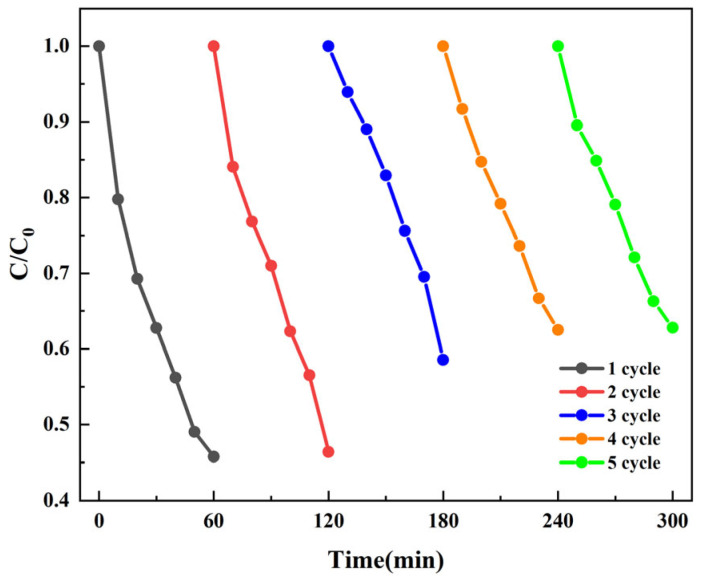
Cycling stability of Ag-CuO/TiO_2_ for TCH degradation under visible light.

The reusability/sustainability of photocatalysts plays an important role in their practical usage, as shown in Figure 9. The selected Ag-CuO/TiO_2_ nanocomposites were collected and washed after one hour of the photocatalytic process and then reused for four additional cycles according to the same procedures. However, a significant decrease in activity was observed in the third cycle, which may result from the aggregation and detachment of silver nanoparticles from CuO/TiO_2_. These features as well as the mass loss during the photodegradation process and the recycle process may contribute to the significant decrease in photocatalytic efficiency in the third cycle. The XRD patterns of the Ag-CuO/TiO_2_ photocatalyst before and after the photocatalytic reaction are shown in Figure S3 (Supplementary Materials).

### 3.3. Electrochemical Measurements

**Figure 10:**
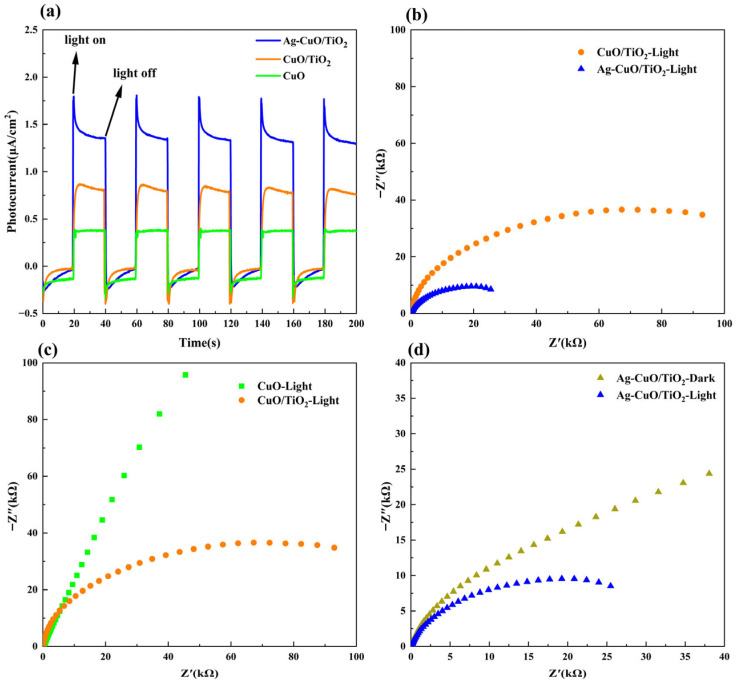
Photoelectrochemical properties of CuO, CuO/TiO_2_ and Ag-CuO/TiO_2_: (**a**) photocurrent responses; (**b**–**d**) electrochemical impedance spectroscopy (EIS).

The transient photocurrent responses of CuO, CuO/TiO_2_, and Ag-CuO/TiO_2_ under visible-light irradiation are shown in Figure 10a. Both CuO/TiO_2_ and Ag-CuO/TiO_2_ exhibit distinct photocurrent signals upon light exposure, with Ag-CuO/TiO_2_ showing the highest photocurrent among the three samples. This enhancement indicates that the incorporation of Ag nanoparticles promotes the separation and migration of photoexcited carriers, thereby improving the photocatalytic performance. In addition, the Nyquist plots under visible-light illumination are presented in Figure 10b–d. Each plot consists of a high-frequency semicircle and a low-frequency inclined line, where the diameter of the semicircle reflects the interfacial charge-transfer resistance (R_t_c). The semicircle of CuO/TiO_2_ is markedly smaller than that of pure CuO, suggesting that the TiO_2_ coating reduces interfacial resistance and facilitates electron transport. The introduction of Ag nanoparticles further decreases the semicircle diameter of Ag-CuO/TiO_2_, indicating enhanced charge-transfer efficiency in the ternary composite. Figure 10d compares the impedance behavior of the Ag-CuO/TiO_2_ electrode under dark and illuminated conditions, showing a smaller semicircle radius under light, which confirms that photogenerated carriers participate in interfacial charge transfer and reduce apparent charge-transfer resistance. These results are consistent with the increased photocurrent, demonstrating that the incorporation of Ag significantly accelerates interfacial electron migration and improves photoinduced charge separation [52,58,59].

### 3.4. Photocatalytic Degradation Mechanism

**Figure 11:**
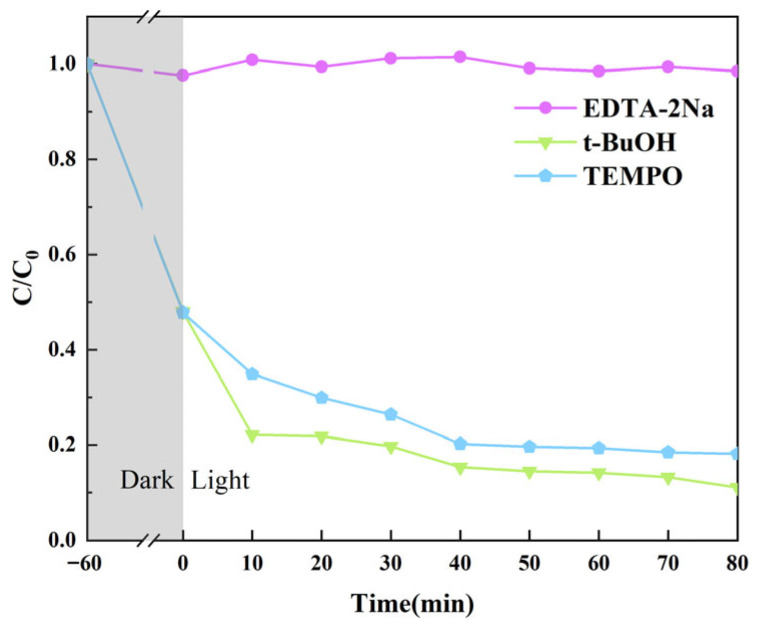
Effect of radical scavengers on TCH degradation over Ag-CuO/TiO_2_ under visible light. EDTA-2Na, TEMPO, and t-BuOH were used to quench h^+^, •O_2^−^_, and •OH, respectively.

Radical scavenging experiments were conducted to identify the reactive species primarily responsible for the degradation of tetracycline (TCH) over Ag-CuO/TiO_2_ under visible light (Figure 11). The presence of EDTA-2Na, a hole-trapping agent, led to a marked decrease in photocatalytic activity, indicating that photogenerated holes are the dominant oxidizing species. In contrast, the addition of TEMPO, a superoxide radical scavenger, caused a moderate decline in degradation efficiency, while t-BuOH, a hydroxyl radical scavenger, exerted little effect, suggesting that hydroxyl radicals play a negligible role in this system [60].

Based on these observations, the photocatalytic mechanism can be represented as follows:

Ag-CuO/TiO_2_ + hν → e_CB^−^_ + h_VB^+^_, (1)

h_VB^+^_ + TCH → Degradation Production, (2)

e_CB^−^_ + O_2_ → •O_2^−^_, (3)

•O_2^−^_ + TCH → Degradation Production, (4)

These results demonstrate that the degradation of TCH is predominantly driven by photogenerated holes, with superoxide radicals contributing to a lesser extent, while hydroxyl radicals are of minimal significance [61,62].

**Figure 12:**
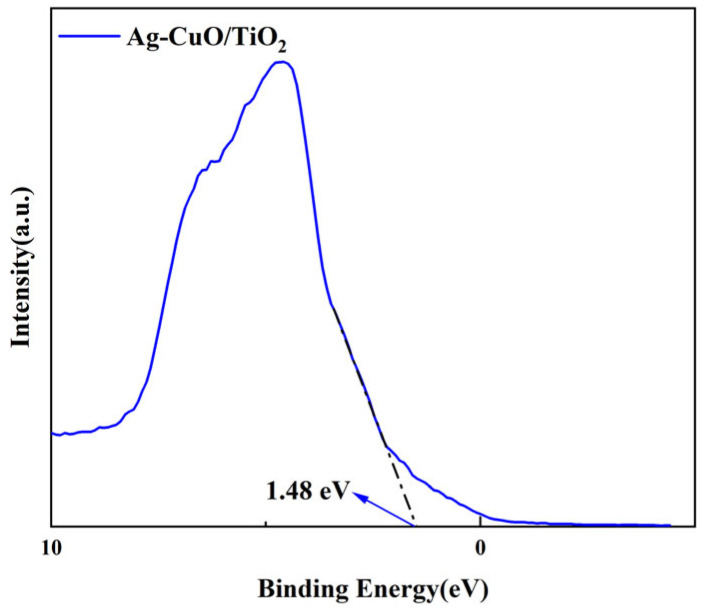
XPS valence band spectra of Ag-CuO/TiO_2_.

To understand this behavior from an electronic structure perspective, the band positions of Ag-CuO/TiO_2_ were estimated using XPS valence band data (Figure 12) in combination with the optical band gap obtained from UV-Vis diffuse reflectance spectra. The valence band maximum determined by linear extrapolation is located at 1.48 eV relative to the Fermi level. The valence band potential versus the normal hydrogen electrode (NHE) was calculated according to

E_VB_ (NHE) = φ + E_VB_ (XPS) − 4.44, (5)

where φ is the spectrometer work function (4.2 eV). The resulting valence band position is therefore +1.24 eV vs. NHE. Using the band gap energy (E_g_ = 1.22 eV), the conduction band potential was derived from

E_CB_ = E_VB_ − E_g_, (6)

giving a value of +0.02 eV vs. NHE [63,64,65].

These energy levels are consistent with the radical scavenging results: the VB is insufficiently positive to oxidize H_2_O or OH^−^ to •OH, and the CB is only slightly negative, limiting the formation of •O_2^−^_. Therefore, direct oxidation by photogenerated holes is thermodynamically favored, whereas •O_2^−^_ contributes as a secondary oxidant and •OH plays a negligible role.

**Figure 13:**
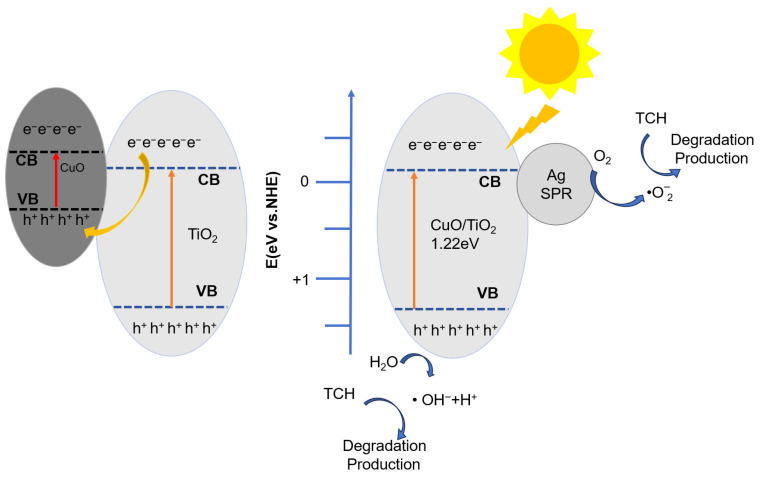
Proposed photocatalytic mechanism of Ag-CuO/TiO_2_ under visible light.

Based on the above analysis, a plausible charge-transfer mechanism under visible-light irradiation is illustrated in Figure 13. Upon illumination, CuO is preferentially excited to generate electron-hole pairs. The interfacial band alignment drives the separation of photogenerated carriers, with electrons transferring towards TiO_2_ and being effectively extracted by Ag nanoparticles, which suppresses electron-hole recombination. A portion of the electrons can react with dissolved oxygen to produce a limited amount of reactive oxygen species, while the holes in the CuO valence band directly oxidize adsorbed TCH molecules and their intermediates. In this system, the primary role of Ag is to facilitate electron extraction and interfacial charge transfer, as well as to enhance visible-light utilization, rather than to alter the dominant oxidation pathway. Therefore, the improved photocatalytic performance of Ag-CuO/TiO_2_ mainly results from efficient charge separation and utilization, with photogenerated holes acting as the principal active species [37,46].

## 4. Conclusions

Ag-CuO/TiO_2_ ternary composites were successfully synthesized and evaluated for the visible-light-driven degradation of tetracycline hydrochloride. The incorporation of TiO_2_ and Ag nanoparticles markedly enhanced charge separation and transfer, as demonstrated by transient photocurrent measurements and electrochemical impedance spectroscopy. Among the samples, Ag-CuO/TiO_2_ exhibited the highest photocatalytic activity, achieving 80.7% degradation of tetracycline within 60 min, with an apparent first-order rate constant of 0.01457 min^−1^. Radical scavenging experiments confirmed that photogenerated holes were the primary reactive species, whereas superoxide and hydroxyl radicals played secondary roles. XPS and UV-Vis analyses indicated valence and conduction band positions of +1.24 eV and +0.02 eV versus NHE, respectively, supporting hole-mediated oxidation. The composites maintained their activity over five consecutive cycles, demonstrating excellent stability. These findings indicate that the synergistic effects of TiO_2_ heterojunction formation and Ag-induced electron extraction significantly improve charge carrier utilization, offering a promising strategy for the design of efficient CuO-based photocatalysts for visible-light-driven organic pollutant degradation.

## Figures and Tables

**Table 1:** Elemental composition and atomic ratios of the Ag-CuO/TiO_2_ composite determined by ICP-OES.

Sample	Cu (wt.%)	Ti (wt.%)	Ag (wt.%)
ACT	27.78	8.07	43.03

**Table 2:** Removal efficiency of tetracycline hydrochloride compared with commercially available Ag-CuO/TiO_2_, Nb_2_O_5_, ZnO, TiO_2_ and Au-Nb_3_O_7_F.

Catalysts	Ag-CuO/TiO_2_	Nb_2_O_5_	ZnO	TiO_2_	Au-Nb_3_O_7_F
C/C_0_ at 60 min	80.7%	6.8%	34.8%	42.1%	50.6%

## Data Availability

The original contributions presented in this study are included in the article/Supplementary Materials. Further inquiries can be directed to the corresponding author.

## Supplementary Materials

The following supporting information can be downloaded at: https://www.mdpi.com/article/10.3390/ma19112189/s1, Figure S1: (a) N_2_ adsorption-desorption isotherms and (b) BJH pore size distribution curves of CuO, CuO/TiO_2_, and Ag-CuO/TiO_2_; Figure S2: UV-Vis absorption spectra of tetracycline over Ag-CuO/TiO_2_ at different irradiation; Figure S3: XRD patterns before and after reaction; Table S1: Elemental composition of the Ag-CuO/TiO_2_ composite determined by EDS.
