# Supplementary material for: Facile Preparation of a Plasmon-Enhanced Ag-CuO/TiO2 for the Efficient Visible-Light-Driven Photodegradation of Tetracycline Hydrochloride
Source: Materials (Basel). 2026 May 22;19(11):2189. doi: 10.3390/ma19112189 (PMC13258318; doi:10.3390/ma19112189)
Supplement: Supplementary file 1 [file materials-19-02189-s001.zip › materials-4255726-supplementary.pdf]

Supplementary Material

Facile Preparation of a Plasmon-Enhanced Ag-CuO/TiO_2 for the Efficient Visible-Light-Driven Photodegradation of Tetracycline Hydrochloride

Lianmin Cui, Li Ren *, Zhi Chen, Benfeng Zhu, Chen Xu and Guoying Wei *

Page S2: N_2 adsorption–desorption isotherms of CuO, CuO/TiO_2, and Ag–CuO/TiO_2

Page S3: Elemental composition of the Ag-CuO/TiO_2 composite determined by EDS analysis

Page S4: UV-Vis absorption spectra of tetracycline over Ag-CuO/TiO_2 at different irradiation times under visible light

Page S5: XRD patterns before and after reaction

Page S6: References

Ⅰ: N_2 adsorption–desorption isotherms of CuO, CuO/TiO$_2$, and Ag–CuO/TiO$_2$

The N_2 adsorption–desorption isotherms of all samples (Fig. S1) display typical type IV behavior. A gradual increase in adsorption occurs at low relative pressures ($P/P_0 < 0.3$), followed by a more pronounced uptake at higher relative pressures ($P/P_0 > 0.8$).The specific surface areas of CuO, CuO/TiO_2, and Ag-CuO/TiO_2 are 13.74, 8.35, and 8.70 $m^2 \cdot g^{-1}$, respectively. Notably, Ag-CuO/TiO_2 exhibits a slightly higher surface area. The pore size distributions (Fig. S1) were calculated from the desorption branch using the BJH method. Pores are primarily distributed in the range of 10-30 nm. The average pore diameters are 19.79 nm for CuO, 28.21 nm for CuO/TiO_2, and 26.06 nm for Ag-CuO/TiO_2, with corresponding pore volumes of 0.068, 0.059, and 0.057 $cm^3 \cdot g^{-1}$.Ag-CuO/TiO_2 exhibits a slightly smaller average pore diameter and a comparable pore volume, while retaining mesoporous structural characteristics[1–3].

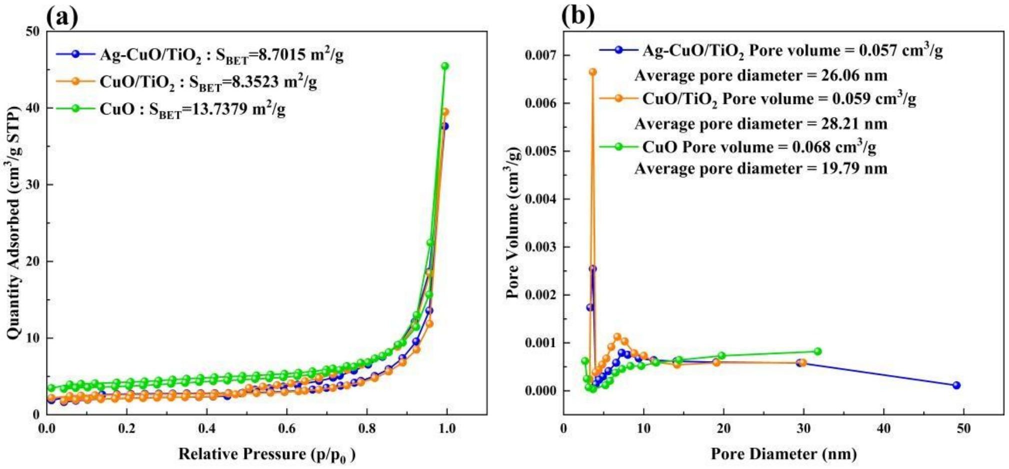

Figure S1.(a) N_2 adsorption-desorption isotherms and (b) BJH pore size distribution curves of CuO, CuO/TiO_2, and Ag-CuO/TiO_2

Ⅱ**: Elemental composition of the Ag-CuO/TiO_2 composite determined by EDS analysis**

Table S1.Elemental composition of the Ag-CuO/TiO_2 composite determined by EDS analysis

**Sample**	Cu (at.%)	Ti (at.%)	Ag (at.%)	O (at.%)
ACT	28.03	7.63	24.43	39.92

III: **UV–vis absorption spectra of tetracycline during photocatalytic degradation**

The UV-Vis absorption spectra of tetracycline during the photocatalytic degradation process are shown in Figure S2. The characteristic absorption peaks gradually decreased in intensity with increasing irradiation time, indicating the progressive degradation of tetracycline molecules.

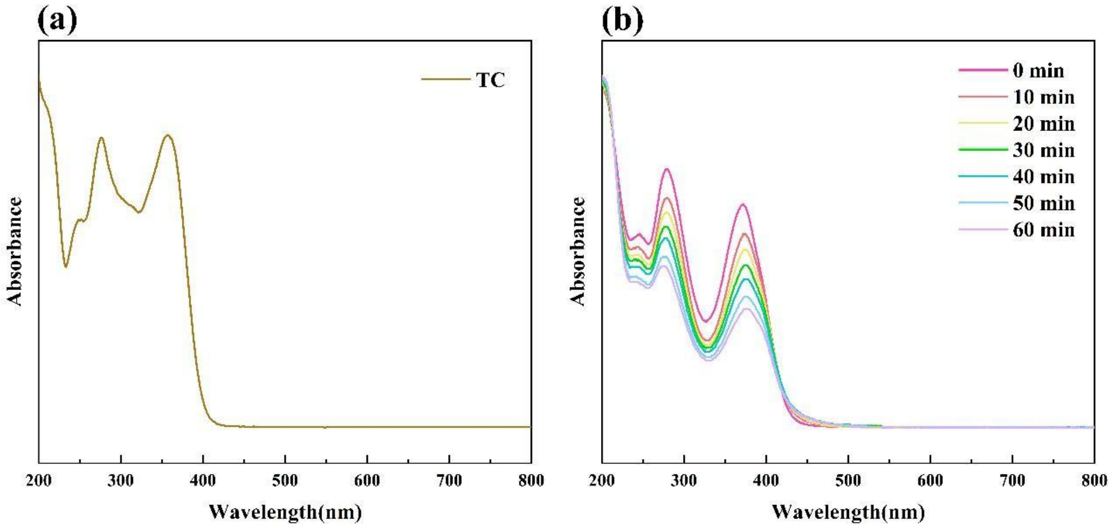

Figure S2.UV-Vis absorption spectra of tetracycline over Ag-CuO/TiO_2 at different irradiation times under visible light

Ⅳ: **XRD patterns of the Ag–CuO/TiO_2 photocatalyst before and after photocatalytic reaction**

Figure S3 shows the XRD patterns of the Ag-CuO/TiO_2 photocatalyst before and after the photocatalytic reaction. Compared with the fresh sample, the main diffraction peaks of the used catalyst remain at nearly the same positions, indicating that the crystal structure is well preserved during the reaction. In addition, the diffraction peaks of the used sample become sharper, suggesting an increase in crystallinity, which may be attributed to slight recrystallization during the cycling process or the subsequent drying treatment. Meanwhile, the slight decrease in photocatalytic performance observed in the cycling test may be ascribed to the gradual accumulation of organic species and their intermediates on the catalyst surface, which partially block the active sites and hinder the reaction[1].

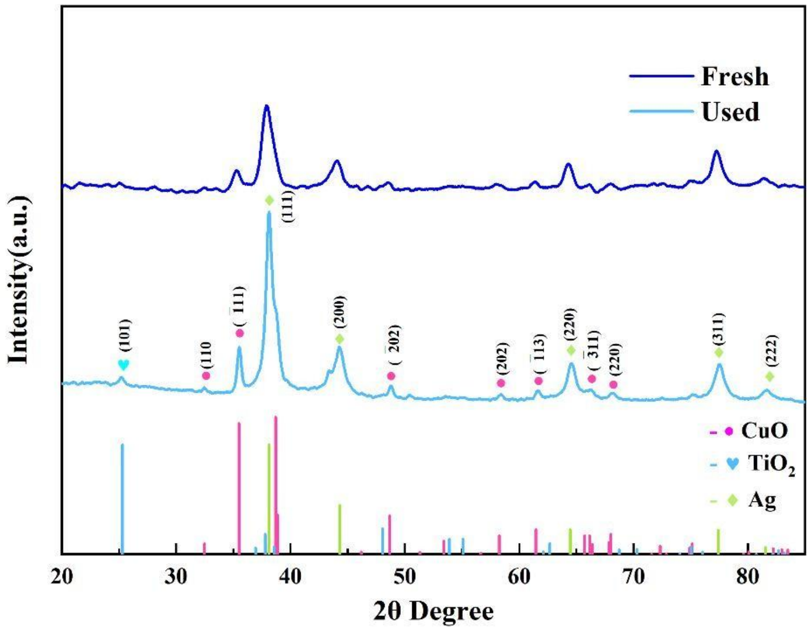

Figure S3.XRD patterns before and after reaction

References

1. Su, Y.; Zeng, Z.; Chen, H.; Lv, Z.; Tan, C.; Chen, C. Preparation and Photocatalytic Degradation Performance of C@CdxMn1−xS to Tetracycline Hydrochloride. *Materials* **2025**, *18*, 1062, doi:10.3390/ma18051062.
2. Cui, W.; Zhang, C.; Li, S.; Liu, Y.; Tian, L.; Li, M.; Zhi, Y.; Shan, S. The Construction of Z-Scheme Heterojunction $ZnIn_2S_4$@CuO with Enhanced Charge Transfer Capability and Its Mechanism Study for the Visible Light Degradation of Tetracycline. *Journal of Colloid and Interface Science* **2024**, *669*, 402–418, doi:10.1016/j.jcis.2024.04.163.
3. Chen, X.; Wu, J.; Wang, X.; Jia, R.; Li, L.; Wang, Y.; Cai, Y.; Chen, Z.; Jin, C.-C.; Wang, X.; et al. Molecule Self-Assembly of Hydrangea-Shaped Hollow O, Cl −codoped Graphite-Phase Carbon Nitride Microspheres for Efficient N-(1,3-Dimethyl Butyl)-N′-Phenyl-p-Phenylenediamine Quinone Photodegradation and Bacteria Disinfection. *Journal of Colloid and Interface Science* **2025**, *683*, 1049–1056, doi:10.1016/j.jcis.2025.01.003.
